# Novel alterations in *IFT172* and *KIFAP3* may induce basal cell carcinoma

**DOI:** 10.1186/s13023-021-02033-7

**Published:** 2021-10-21

**Authors:** Shoko Onodera, Nana Morita, Yuriko Nakamura, Shinichi Takahashi, Kazuhiko Hashimoto, Takeshi Nomura, Akira Katakura, Kenjiro Kosaki, Toshifumi Azuma

**Affiliations:** 1 Department of Biochemistry, Tokyo Dental College, 2-9-18, Kanda Misakichou, Chiyoda, Tokyo 101-0061 Japan; 2 Department of Oral Medicine and Hospital Dentistry, Tokyo Dental College, 5-11-13, Sugano, Ichikawa, Chiba 272-8513 Japan; 3 Department of Dentistry, Oral and Maxillofacial Surgery, Tama-Hokubu Medical Center, Tokyo Metropolitan Health and Medical Treatment Corporation, 1-7-1, aoba-cho, Higashimurayama, Tokyo 189-8511 Japan; 4 Department of Oral Oncology, Oral and Maxillofacial Surgery, Tokyo Dental College, 5-11-13, Sugano, Ichikawa, Chiba 272-8513 Japan; 5 Department of Dermatology, Tokyo Dental College Ichikawa General Hospital, 5-11-13, Sugano, Ichikawa, Chiba 272-8513 Japan; 6 Division of Surgical Pathology, Clinical Laboratory, Tokyo Dental College Ichikawa General Hospital, 5-11-13, Sugano, Ichikawa, Chiba 272-8513 Japan; 7 Department of Oral and Maxillofacial Surgery, Tokyo Dental College, 2-9-18, Kanda Misakichou, Chiyoda, Tokyo 101-0061 Japan; 8 Center for Medical Genetics, Keio University School of Medicine, 2-15-45, Mita, Minatoku, Tokyo 108-8345 Japan

**Keywords:** Gorlin syndrome, Nevoid basal cell carcinoma, Basal cell carcinoma, Odontogenic keratocyst, Exome sequence, Gene mutation, Hedgehog signaling

## Abstract

**Background:**

Basal cell carcinoma (BCC) is the most commonly occurring neoplasm in patients with Gorlin syndrome. It is widely accepted that multiple basal cell carcinomas simultaneously develop in middle-aged patients with this syndrome. However, the presence of driver genes other than the *PTCH1* in Gorlin syndrome has not been explored. This study aimed to identify common gene mutations other than *PTCH1* in simultaneously occurring basal cell carcinomas in patients with Gorlin syndrome via exome sequencing analysis.

**Methods:**

Next-generation sequencing analysis was performed using four basal cell carcinoma samples, one dental keratinocyte sample, and two epidermoid cyst samples, which were surgically resected from one patient with Gorlin syndrome on the same day.

**Results:**

Overall, 282 somatic mutations were identified in the neoplasms. No additional somatic mutations in *PTCH1*, *PTCH2*, *TP53*, and *SMO* were identified. However, enrichment analysis showed that multiple genes, such as *IFT172* and *KIFAP3,* could regulate ciliary functions important for Hedgehog signaling.

**Conclusion:**

The development of BCCs in patients with Gorlin syndrome may be triggered by mutations that cause substantial dysfunction of cilia.

## Background

Basal cell carcinoma (BCC) (MIM 109400) is the most commonly reported skin neoplasm, with an estimated overall lifetime risk of 20–30% [1]. The yearly incidence of BCC in Japan and the European Union is approximately 3.34 and 32.05 per 100,000 people, respectively [2, 3]. While it is a slow-growing, rarely metastasizing tumor that occurs primarily on sunburned skin, it can disfigure local tissues if left untreated or improperly treated.

Gorlin syndrome, also known as nevoid basal cell carcinoma syndrome, is a hereditary disease that was characterized by Gorlin and Goltz in the 1960s, and is associated with germline mutations in the hedgehog receptor gene *PTCH1,* along with the exhibition of significant associations with BCC [4]. Genetic analysis of this syndrome has contributed markedly to the understanding of genetic changes that occur in BCC. It is well accepted that BCCs in Gorlin syndrome and sporadic BCCs exhibit aberrant activation of the Hedgehog (Hh) pathway. This is caused by genetic inactivation of *PTHC1* or mutational activation of SMO, a key Hh signaling molecule that promotes the activity and nuclear localization of GLI transcription factors [5, 6]. Since BCCs mostly occur in middle-aged patients with Gorlin syndrome, Hh activation seems to be necessary for its development.

It has been well documented that alterations in *TP53* are commonly observed in sporadic BCCs [7], with more than half of the cases presenting with *TP53* mutations along with activation of Hh [8]. However, there are few reports describing *TP53* mutations in BCC of patients with Gorlin syndrome. In fact, there are no clinical reports available on the identification of genes other than *PTCH1* that drive the development of BCC in patients with Gorlin syndrome. A unique feature of Gorlin syndrome BCC development is that BCC usually occurs in middle-aged and older patients rather than younger patients, and they usually prone to occur in multiple lesions simultaneously. Much remains unknown about whether additional mutations in genes other than those in *PTCH1* exist.

Previously, we observed via exome sequence analysis that two out of four patients with Gorlin syndrome exhibited mutations in both the *PTCH1* and *PTCH2* genes [9]. Notably, one of the patients in this study did not demonstrate the development of BCC until the age of 50 years despite significant constitutive activation of Hh signaling caused by the double mutations in *PTCH1* and *PTCH2*. Simultaneously occurring multiple BCCs were observed in this patient. In this study, we treated the cases via excision and performed extraction of their DNA for genomic analysis. We successfully excised and extracted the genomes of four different BCCs, two skin cysts, and one odontogenic keratocyst (OKC) on the same day. Finally, we performed exome sequencing and identified genomic mutations that had not been previously reported.

## Results

### Confirmation of each tumor and cyst

**Fig. 1:**
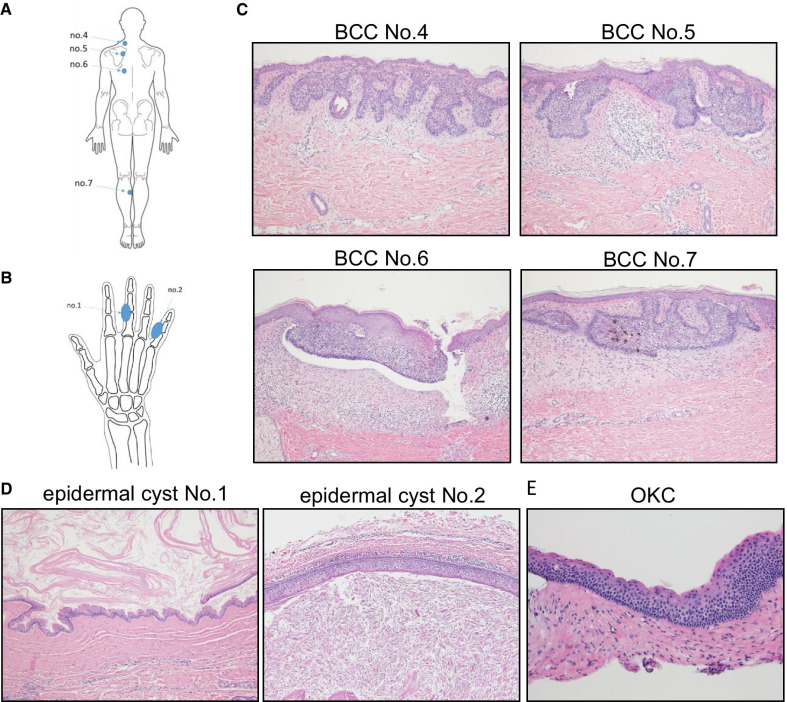
Histologic analysis of the tumors and cysts. **a** A schema of the positions of the BCCs (numbers are related to **c**). **b** A schema of the positions of the epidermal cysts (numbers are related to **d**). **c** Histologic analysis of the basal cell carcinomas (BCCs). Each sample is represented in **a**. **d** Histologic analysis of the epidermal cysts. Each sample is represented in **b**. **e** Histologic analysis of the odontogenic keratinocyte

One male patient met four of the major criteria of the Gorlin syndrome, with the existence of *PTCH1* and *PTCH2* mutations in his normal oral tissue [9]. Eight tumors were individually removed from skin lesions at different sites to identify additional mutations. Dermal cyst samples were obtained from the third and fifth fingers, and mandibular OKC samples were collected. Genomic DNA samples from the eight tumors and three cyst samples were extracted and evaluated. Four of the eight samples met the next-generation sequencing criteria and were subsequently analyzed using exome sequencing analysis. Three of the four samples were derived from the patient's back (Fig. 1a and c, no. 4, 5, 6), while the other was derived from the back of the patient’s lower leg (Fig. 1a and c, no. 7).

The following characteristic findings of BCC were observed in all collected tumor samples: (1) existence of basal cell-like atypical cells that proliferate like buds, (2) presence of a gap at the interface between the connective tissue and the basal cell layer (Fig. 1c, no. 6), (3) an arrangement of the basal tumor layer in a palisading pattern, and (4) melanin pigmentation (Fig. 1c, no.7). A pathologist examined and identified the four tumors subjected to exome sequencing as superficial basal cell carcinomas, characterized by erythematous patches ranging from a few millimeters to > 10 cm [10].

As shown in Fig. 1b and d, the following two characteristic findings were observed in the histological observation of the skin cyst: (1) a highly keratinized and normalized stratified squamous epithelium lining of the cyst, and (2) layered keratin. Based on these observations, the two cysts were diagnosed as epidermoid cysts.

Figure 1e shows the following two observed characteristics in the OKC: (1) a parakeratotic stratified squamous epithelium lining of the cyst, and (2) a palisade arrangement of the basal layer of the lined epithelium. Based on these findings, the cyst was diagnosed as an OKC.

As described above, histological analysis confirmed the four tumors as basal cell carcinomas (BCCs) and the cysts as epidermoid cysts.

### Somatic mutations in hedgehog-related genes are not observed in BCCs

**Fig. 2:**
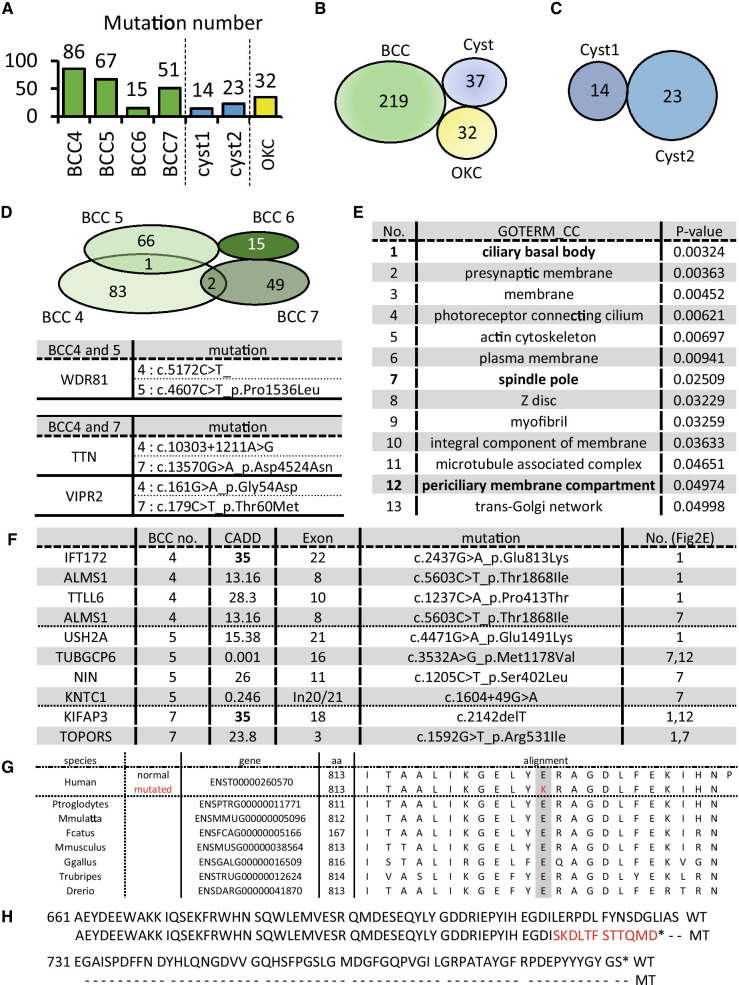
Somatic mutations of the tumors and cysts. **a** Somatic mutations of the tumors in causative genes. **b** Mutation numbers of each sample. The numbers above each bar indicate the number of mutations. **c** A Venn diagram illustrating the mutation genes in the BCCs (green), cysts (blue), and OKC (yellow). The numbers indicate the total mutations in each group. **d** A Venn diagram illustrating the mutation genes in each BCC. The numbers indicate the total number of mutations in each BCC. The mutations of the genes indicated on the right indicate the associations between BCC4 and BCC5 as well as BCC4 and BCC7. **e** A list of Gene Ontology enrichment terms with *P*-values above 0.05 in the additional mutations. Bold characters indicate relation to cilia components. **f** A list of genes containing cilia-related GO terms, as shown in **e**. The number refers to the GO term in **e** containing the genes. **g** The conservation of the mutational regions of *IFT172*. **h** The wild-type and mutant forms of KIFAP3

Next, the genomes of the tissue samples were analyzed using next-generation sequencing. A total of 282 somatic coding mutations were confirmed using exome analysis. As previously reported by our group, all samples obtained from this patient showed genomic mutations in both *PTCH1* and *PTCH2* genes [9]. In addition to the previously reported mutations, we assessed for new mutations occurring in BCC driver genes, such as *P53*, or genes particularly known as hedgehog-related genes, such as *SMO* or *SUFU*. However, no additional mutations were identified. Thus, we evaluated for other mutations in the BCCs and cysts and found that BCCs exhibited more somatic mutations than the cysts or OKC (Fig. 2a). There were no identical mutations in the BCCs or cysts (Fig. 2b, c). However, multiple mutations of the identical gene were observed in the BCC tumor samples 4, 5, and 7 (Fig. 2d; upper). However, no common mutations were detected among these mutations (Fig. 2d; lower).

Based on these results, a common gene mutation was not identified among the simultaneously occurring basal cell carcinomas in the patients with Gorlin syndrome. Furthermore, these mutations were not identified in the skin cysts that occurred concurrently.

### Mutational signature of BCC

Since no additional common mutations were observed in the BCC samples, additional driver genes responsible for BCC development were not identified [15, 16]. Thus, the detected mutant genes were analyzed and classified according to their protein functions and other factors. A pathway enrichment analysis using DAVID identified multiple Gene Ontology (GO) categories significantly enriched in the altered genes (Fig. 2e). Notably, the 216 modified genes of BCC have been classified by the GO terminology and reported to be involved in the basal ciliary body (GO: 0036064), spindle pole (GO: 0000922), and periciliary membrane compartment (GO: 1990075) (Fig. 2f), which are related to cilium component proteins. Each BCC in this study, except for no. 6, exhibited genetic mutations in these genes. Variants were annotated using combined annotation dependent depletion (CADD), a method that involves the integration of functional annotations, conservation, and gene-model information into a single metric [11, 12]. The CADD scores of *IFT172* and *KIFAP3* exceeded 30, suggesting that these mutations were within the top 0.1% of the mutations assumed to be the most harmful. (Fig. 2f) [12]. However, data on the mutations in these genes were not found in Exome Aggregation Consortium (ExAC) [13, 14]. Analysis involving the use of MUTATION TASTER2, an in silico protein function prediction program, identified *IFT172* (Glu813Lys) as a possible cause of the disease [15, 16]. PhyloP and PhastCons, which are used to determine the extent of phylogenetic nucleotide sequence conservation, yielded values of 4.788 and 1, respectively, indicating that the genomic region of Glu813Lys was well conserved (Fig. 2g) [17, 18]. Additionally, 2142delT in *KIFAP3* induced a frameshift mutation, causing amino acid changes that produced shorter proteins than the wild-type (Fig. 2h). The CADD score of *TTLL6*, *NIN*, and *TOPORS* exceeded 20, indicating that they were within the top 1% of the mutations assumed to be harmful (Fig. 2f) [12].

## Discussion

Tumors transform from benign to malignant lesions by acquiring a series of mutations. Several malignancies are thought to be caused by 2–8 driver gene mutations. In patients with Gorlin syndrome, mutations are found in *PTCH1*, the driver gene for non-syndromic BCC. Additional mutations in other driver genes of BCC would increase the chances of tumor progression to malignancy. We previously reported the presence of mutations in *PTCH2* and *BOC,* alongside *PTCH1,* in four unrelated patients with Gorlin syndrome patient via exome sequencing. These identified genes were Hh receptor molecules, similar to *PTCH1* [9]. This is the first evidence reported on multi-layered mutations in the Hh pathway that cause alteration in the activation levels of Hh signals.

One of the patients described in a previous study was included as a subject of the present study. This patient harbored germline mutations in *PTCH1*, *PTCH2*, and *PIK3CA*, which were hypothesized to be driver gene mutations. *PTCH1* and *PTCH2* mutations that cause overactivation of the Hh pathway are beneficial for the selective proliferation of normal basal cells.

Interestingly, multiple basal cell carcinomas in patients with Gorlin syndrome tend to occur simultaneously; however, the mechanism and driver gene mutations underlying the simultaneous development of BCCs remain unclear. Thus, we investigated the presence of additional driver mutations. While several additional somatic mutations were observed in every sample, no common driver gene mutations were detected among them.

Next, we examined whether the mutated genes shared functional similarities. We used combined annotation dependent depletion (CADD) to integrate diverse annotations into single measures for each variant objectively. We found that the mutated genes were enriched in the major cilia components in three of the four samples. Since ciliary functions are fundamentally involved in the Hh signaling pathway, and as several studies have shown that genetic mutations in cilia may contribute to tumor formation, these cilia component gene alterations may have contributed to the malignant transformation of BCCs. We determined CADD scores of 30 or higher in the mutations observed in *IFT172* and *KIFAP3*, which encode ciliary proteins, indicating that they were within the top 0.1% deleterious gene mutations of the human genome [12]. *IFT172* encodes a major intraflagellar transport (IFT) protein that is essential for ciliogenesis [23], and disruption of this gene results in a complete lack of primary cilia [20]. The anterograde transport from the base to the tip of IFT is powered by two conserved, dedicated microtubule motors, namely a plus-end-directed heterotrimeric kinesin-2 complex, KIF (KIF3a, KIF3b), and KIF-associated protein 3 (KIFAP3) [21]. Recent studies have shown that KIFAP3 directly establishes interactions with GLI proteins in vitro [22], and disruption of its gene in mice results in the development of BCC-like malignant neoplasia [23].

Primary cilia play prominent roles in modulating mammalian Hh signaling [24]. Once the hedgehog protein binds to the PTCH receptor, SMO accumulates in the primary cilium via translocation caused by the complex formation of arrestin and KIF3A. SMO activation results in the concentration and nuclear localization of SUFU and GLI1 in the cilia. Thus, the trans-localization of SMO is critical for Hh signal transduction [25]. Furthermore, ciliary function plays an important role in the tumorigenesis of medulloblastoma and basal cell carcinoma, two major tumors observed in Gorlin syndrome [25]. In mice, activation of SMO resulted in the development of medulloblastoma or basal cell carcinoma, which was blocked by inhibition of cilia formation [23]. Therefore, the mutations in the abovementioned genes in this patient may have contributed to the malignant transformation of BCCs. It is also noteworthy that primary ciliary dyskinesia, an autosomal recessive genetic condition that causes malfunction of cilia in the respiratory system, has been associated with cancer [26].

Recent studies suggest that non-coding RNAs and epigenetic factors may also be intrinsically involved in carcinogenesis. Even if the same gene mutation has not been confirmed between multiple simultaneous tumors, non-coding RNA expression in certain tumors in patients with Gorlin syndrome may have contributed to BCC co-occurrence. In fact, the involvement of miRNAs has also been reported in BCC. Heffelfinger et al. showed that 20 mature miRNAs exhibited differential expression in two subtypes of BCC, indicating that non-coding RNAs (ncRNAs) might alter BCC properties [27]. miRNA-451a expression was significantly reduced in human BCC tissues. Downregulation of miRNA-451a expression was also confirmed in the BCC mouse model, while overexpression of miRNA-451a in tumor cells markedly suppressed cell growth through G1 cell cycle arrest [28]. These findings indicate that ncRNA and epigenetic functions may regulate the molecular pathogenesis of BCC.

## Conclusions

In this study, in addition to the PTCH1 mutation, various types of mutations were found in the BCCs of one patient with Golrin syndrome. These mutations found in the BCCs may result in substantial ciliary dysfunction and IFT, which may cause BCC formation; for further validation, further investigations are warranted.

## Methods

### Ethics statement

Written informed consent was obtained from the participant in the present molecular genetics study. The study was approved by the ethics committee for clinical research at the Tokyo Dental College Suidoubashi Hospital and Ichikawa General Hospital (Tokyo, Japan) (no. 527, no. 575, and I 15-78RII). Furthermore, it complied with the tenets of the declaration of Helsinki.

### Pathology

Eight and three skin lesions suspected of harboring BCC and cyst, respectively, were found, and all lesions were surgically removed. Surgically removed samples were cut into two pieces, and one portion was processed for pathological diagnosis. After conducting fixation in 10% paraformaldehyde, the samples were embedded in a paraffin block. Sections of fixed tissues (3 um) mounted on glass slides were assayed by conducting hematoxylin and Eosin (H&E) staining according to the general method. We performed extraction of genomic DNA for exome sequencing from remaining samples using conventional proteinase K treatment-SDS treatment followed by phenol-chloroform treatment or by using the Easy-DNA™ gDNA Purification Kit (Thermo Fisher Scientific). Two methods were used according to the size of the sample. Briefly, the samples were fixed by liquid nitrogen and homogenized to a small sample. The samples were introduced into the cell lysate buffer (20mg/ml proteinase K and 10%SDS buffer in TE buffer) and incubated at 56 °C until the samples were dissociated. Supernatant from dissociated samples was added to the phenol chloroform mixture, and genomic DNA extraction was performed. The purification of genomic DNA was conducted using the basic ethanol precipitation method.

### Sequencing and data analyses

Genomic DNA extraction was performed for exome sequencing using conventional proteinase K-SDS treatment, followed by phenol–chloroform treatment or by using the Easy-DNA™ gDNA Purification Kit (Thermo Fischer Scientific). These were fragmented using Covaris (Covaris, Woburn, MA, USA). The sizes of the library fragments were approximately 200–250 bp. Enrichment of coding exons was performed using the Sure Select XT Human All Exon v5 kit (Agilent Technologies, California, USA) to generate exome libraries. Paired-end (2X101 bp) DNA libraries were sequenced using the Hiseq 4000 sequencer obtained from Macrogen (South Korea).

### Validation analysis of mutations

Mapping of the sequence reads to the human reference genome (GRCh37) was performed using the Burrows-Wheeler Aligner (BWA) [29] and Genome Analysis Tool Kit (GATK) [30], following the best-practice guidelines packaged in the integrated analysis suite variant tools [31]. Variant calling was accomplished using multiple callers, including the GATK [30]. The data on called variants were annotated using SnpEff [32].

## Abbreviations

BCC: Basal cell carcinoma
Hh: Hedgehog
OKC: Odontogenic keratocyst
GO: Gene ontology
CADD: Combined annotation dependent depletion
KIFAP3: KIF-associated protein 3
ncRNAs: Non-coding RNAs

## Declarations

### Availability of data and materials

The datasets generated and analyzed during the current study are available in the DDBJ databank of Japan (accession number: JGA 000099).

## References

[1] Bulliard J-L, Panizzon RG, Levi F (2009). Epidemiology of epithelial skin cancers. Rev Med Suisse.

[2] Tamaki T, Dong Y, Ohno Y, Sobue T, Nishimoto H, Shibata A (2014). The burden of rare cancer in Japan: application of the RARECARE definition. Cancer Epidemiol.

[3] Gatta G, van der Zwan JM, Casali PG, Siesling S, Dei Tos AP, Kunkler I (2011). Rare cancers are not so rare: the rare cancer burden in Europe. Eur J Cancer.

[4] Gorlin RJ, Goltz RW (1960). Multiple nevoid basal-cell epithelioma, jaw cysts and bifid rib. A syndrome. N Engl J Med.

[5] Hahn H, Wicking C, Zaphiropoulos PG, Gailani MR, Shanley S, Chidambaram A (1996). Mutations of the human homolog of drosophila patched in the nevoid basal cell carcinoma syndrome. Cell.

[6] Raleigh DR, Reiter JF (2019). Misactivation of Hedgehog signaling causes inherited and sporadic cancers. J Clin Invest.

[7] Giglia-Mari G, Sarasin A (2003). TP53 mutations in human skin cancers. Hum Mutat.

[8] Benjamin CL, Ananthaswamy HN (2007). p53 and the pathogenesis of skin cancer. Toxicol Appl Pharmacol.

[9] Onodera S, Saito A, Hasegawa D, Morita N, Watanabe K, Nomura T (2017). Multi-layered mutation in hedgehog-related genes in Gorlin syndrome may affect the phenotype. PLoS ONE.

[10] Rubin AI, Chen EH, Ratner D (2005). Basal-cell carcinoma. N Engl J Med.

[11] Rentzsch P, Witten D, Cooper GM, Shendure J, Kircher M (2019). CADD: predicting the deleteriousness of variants throughout the human genome. Nucl Acids Res.

[12] Kircher M, Witten DM, Jain P, O’Roak BJ, Cooper GM, Shendure J (2014). A general framework for estimating the relative pathogenicity of human genetic variants. Nat Genet.

[13] Lek M, Karczewski KJ, Minikel EV, Samocha KE, Banks E, Fennell T (2016). Analysis of protein-coding genetic variation in 60,706 humans. Nature.

[14] 1000 Genomes Project Consortium, Auton A, Brooks LD, Durbin RM, Garrison EP, Kang HM, et al. A global reference for human genetic variation. Nature. 2015;526:68–74. 10.1038/nature15393 PMC4750478 26432245

[15] Schwarz JM, Rödelsperger C, Schuelke M, Seelow D (2010). MutationTaster evaluates disease-causing potential of sequence alterations. Nat Methods.

[16] Schwarz JM, Cooper DN, Schuelke M, Seelow D (2014). MutationTaster2: mutation prediction for the deep-sequencing age. Nat Methods.

[17] Pollard KS, Hubisz MJ, Rosenbloom KR, Siepel A (2010). Detection of nonneutral substitution rates on mammalian phylogenies. Genome Res.

[18] Cooper GM, Stone EA, Asimenos G, NISC Comparative Sequencing Program, Green ED, Batzoglou S, et al. Distribution and intensity of constraint in mammalian genomic sequence. Genome Res. 2005;15:901–13. 10.1101/gr.3577405 PMC1172034 15965027

[19] Wang Q, Taschner M, Ganzinger KA, Kelley C, Villasenor A, Heymann M (2018). Membrane association and remodeling by intraflagellar transport protein IFT172. Nat Commun.

[20] Iomini C, Babaev-Khaimov V, Sassaroli M, Piperno G (2001). Protein particles in Chlamydomonas flagella undergo a transport cycle consisting of four phases. J Cell Biol.

[21] He M, Agbu S, Anderson KV (2017). Microtubule motors drive hedgehog signaling in primary cilia. Trends Cell Biol.

[22] Carpenter BS, Barry RL, Verhey KJ, Allen BL (2015). The heterotrimeric kinesin-2 complex interacts with and regulates GLI protein function. J Cell Sci.

[23] Wong SY, Seol AD, So P-L, Ermilov AN, Bichakjian CK, Epstein EH (2009). Primary cilia can both mediate and suppress Hedgehog pathway–dependent tumorigenesis. Nat Med.

[24] Eggenschwiler JT, Anderson KV (2007). Cilia and developmental signaling. Annu Rev Cell Dev Biol.

[25] Corbit KC, Aanstad P, Singla V, Norman AR, Stainier DYR, Reiter JF (2005). Vertebrate smoothened functions at the primary cilium. Nature.

[26] Yoshida J, Tsuneyoshi M, Nakamura K, Murakami T, Akamine Y (1986). Primary ciliary dyskinesia with transverse colon carcinoma. Am J Clin Pathol.

[27] Heffelfinger C, Ouyang Z, Engberg A, Leffell DJ, Hanlon AM, Gordon PB (2012). Correlation of global microRNA expression with basal cell carcinoma subtype. G3 Genes Genomes Genet.

[28] Maturo MG, Rachakonda S, Heidenreich B, Pellegrini C, Srinivas N, Requena C (2020). Coding and noncoding somatic mutations in candidate genes in basal cell carcinoma. Sci Rep.

[29] Li H, Durbin R (2009). Fast and accurate short read alignment with Burrows-Wheeler transform. Bioinformatics.

[30] McKenna A, Hanna M, Banks E, Sivachenko A, Cibulskis K, Kernytsky A (2010). The genome analysis toolkit: a MapReduce framework for analyzing next-generation DNA sequencing data. Genome Res.

[31] Sanlucas FA, Wang G, Scheet P, Peng B (2012). Integrated annotation and analysis of genetic variants from next-generation sequencing studies with variant tools. Bioinformatics.

[32] Yamada M, Suzuki H, Shiraishi Y, Kosaki K (2019). Effectiveness of integrated interpretation of exome and corresponding transcriptome data for detecting splicing variants of genes associated with autosomal recessive disorders. Mol Genet Metab Rep.

